# Patterns of the Health and Economic Burden of 33 Rare Diseases in China: Nationwide Web-Based Study

**DOI:** 10.2196/57353

**Published:** 2024-08-27

**Authors:** Jiazhou Yu, Shanquan Chen, Huanyu Zhang, Shuyang Zhang, Dong Dong

**Affiliations:** 1 Jockey Club School of Public Health and Primary Care The Chinese University of Hong Kong Sha Tin China (Hong Kong); 2 International Centre for Evidence in Disability Faculty of Epidemiology and Population Health London School of Hygiene & Tropical Medicine London United Kingdom; 3 Shenzhen Research Institute The Chinese University of Hong Kong Shenzhen China; 4 Clinical Big Data Research Center The Seventh Affiliated Hospital Sun Yat-sen University Shenzhen China; 5 Department of Cardiology Peking Union Medical College and Chinese Academy of Medical Science Beijing China

**Keywords:** rare disease, burden, quality of life, economic, pattern, China

## Abstract

**Background:**

Rare diseases (RDs) affect millions of individuals collectively worldwide, contributing to significant burdens on patients and families in various aspects. However, there is a lack of evidence on the underlying patterns of burdens among diverse RDs for informing targeted social and health policies to address the unmet needs of this vulnerable population.

**Objective:**

This study aimed to examine the underlying patterns of the health and economic burden of 33 different RDs in China and identify the potential determinants.

**Methods:**

A nationwide internet-based cross-sectional survey was conducted in China between 2019 and 2020. Physical and mental health burden was measured by health-related quality of life. Economic burden was evaluated based on the proportions of direct medical, direct nonmedical, and indirect costs relative to household income. We used cluster analysis to identify patterns of health and economic burdens and conducted multinomial logistic regression to explore potential predictors of cluster membership.

**Results:**

The study included 8454 adults and 8491 children affected by 33 RDs. The following 3 clusters were identified: “extremely high burden” (representing 92/8454, 1.1% and 19/8491, 0.2% of adult and pediatric patients, respectively), “overall high burden” (5933/8454, 70.2% and 4864/8491, 57.3%, respectively), and “overall low burden” (2429/8454, 28.7% and 3608/8491, 42.5%, respectively). Wilson disease, Marfan syndrome, and Langerhans cell histiocytosis more likely resulted in an “extremely high burden” than others. Poverty was significantly associated with being in this extremely high burden group. Diseases causing neuromuscular symptoms and requiring long-term treatment (eg, amyotrophic lateral sclerosis, spinocerebellar ataxia, and Dravet syndrome) were prevalent in the “overall high burden” group. Key predictors of this group included older age, lower socioeconomic status, diagnostic delay, and comorbidity.

**Conclusions:**

This study provides novel and valuable evidence on the burden of RDs in developing regions like China. The findings reveal significant disparities in the impact of RDs, emphasizing the need for targeted health care interventions and policies.

## Introduction

Rare diseases (RDs) refer to health conditions that affect a small number of people compared to other common diseases in a population, and they are often genetically based, debilitating, and associated with enormous disease burden [1]. Currently, there is no international consensus on the definition of an RD [2]. It has been defined as a condition affecting no more than 5 in 10,000 people in the European Union [3] and a condition affecting less than 200,000 individuals in the United States [4]. Notably, although individually rare, it is estimated that overall 263-446 million individuals are living with RDs globally [5].

RDs are predominantly chronic progressively incapacitating conditions that significantly impair both physical and mental health, often leading to reduced life expectancies. Although individuals with different RDs exhibit distinct symptoms, they commonly face similar challenges, including delayed diagnosis, lack of treatment, limited access to appropriate health care and social care services, insufficient information and support, and substantial financial burdens involving out-of-pocket medical costs and indirect costs due to productivity losses [6-9]. In 2019, 11.5 million US children and adults affected by 379 RDs presented a collective direct medical cost of US $449 billion, a direct nonmedical cost of US $73 billion, and an indirect cost of US $437 billion, exceeding the costs of some of the most expensive chronic diseases studied in the United States [10]. Therefore, it is crucial to consider RDs as a unique disease category from the perspective of health equity and for the purpose of policy advocacy. As such, in 2021, the United Nations (UN) unanimously passed a resolution for people living with RDs, calling for recognizing and addressing the needs of RD patients and their families [11]. Identifying the specific challenges faced by this group and addressing their unmet needs as a vulnerable community are essential steps in advancing the 2030 Agenda for Sustainable Development and fulfilling the UN’s commitment to “leave no one behind” [11].

To effectively address the challenges through collective advocacy and policy-making, it is imperative to generate more empirical evidence that reflects the burden of RDs as a whole. Existing studies that consider different RDs collectively have quantified the cost [12-17] and measured the impact on overall well-being [18-20]. However, there is a lack of research on the underlying patterns of the burdens of RDs, which can serve as important evidence for the development of services and social and health policies to alleviate the unmet needs of RD populations. To date, RDs are typically classified by affected systems from a clinical perspective. However, similar to general chronic conditions where underlying homogeneous patterns exist regardless of the symptoms or affected systems [21,22], a collective evaluation of RDs may reveal distinct burden profiles among different sociodemographic or disease subgroups. In practice, given the relatively limited resources for RDs compared to other common chronic conditions, it is critical to identify vulnerable population groups and priority conditions among the diverse RDs for more effective resource allocation. Classifying RDs by focusing on the consequences of the disease from a patient perspective can help identify RDs with similar burdens that should receive equitable prioritization in resource allocation, regardless of the affected system.

In China, it is estimated that 16.8 million individuals, including 11.8 million children, are currently living with RDs, resulting in significant burdens on health care systems and the society [23]. To date, China has not yet passed any legislation on RDs or orphan drugs, but several policies and national strategies have been implemented since 2015, including publishing China’s National Lists of Rare Diseases, investing in research on RDs, establishing national databases to collect RD cases, formulating standardized treatment protocols for clinicians, encouraging innovation of RD drugs, and prioritizing new drugs to treat RDs and children for review and approval [24]. Despite ongoing efforts, the health and social policies for RDs in China fall behind those in the United States, European Union, and Japan, and thus, the burdens are inadequately addressed [25,26]. A 2018 survey reported that 42% of Chinese patients with RDs registered with a national organization had not received any treatment for various reasons, and the majority of the remaining patients failed to receive timely and adequate treatment [27]. Although patients with certain conditions, such as Pompe disease, can obtain partial reimbursement for medical expenses through basal medical insurance in China, the effect was estimated to be negligible in relieving the economic burden on patients and families [28]. A national survey reported that less than 1.3% of patients could completely cover their annual medical costs with their household income [29]. Moreover, evidence suggests that individuals with RDs tend to experience misdiagnosis and impaired physical function, which can contribute to increased care-taking burden within families, mental distress, and unemployment [30,31]. Nonetheless, few resources are available to support the mental health and social well-being challenges faced by patients, leaving considerable unmet needs in this population [30]. To better understand the scope and magnitude of challenges encountered by RD populations and to inform targeted interventions and more effective resource allocation, this study aimed to examine the pattern of the health and economic burdens of RDs in China among adult and pediatric populations, using data from a nationwide survey.

## Methods

### Study Design and Data Collection

This study was a nationwide, internet-based cross-sectional survey conducted in China between August 2019 and January 2020. The study was conducted in collaboration with the China Alliance for Rare Diseases, Peking Union Medical College Hospital, Illness Challenge Foundation, and Chinese University of Hong Kong.

Data were collected through an online questionnaire, which was uploaded to the largest online survey platform in China with a high security level and functions that allow for quality enhancement of responses. Previous research has suggested that internet-based surveys generate a higher response rate than mail surveys [32] and more accurate outcomes than telephone surveys, particularly with relatively sensitive topics [33]. In this study, 2 versions (self-reporting and proxy-reporting) of questionnaires were designed with the same measures but with questions differently phrased to generate more accurate responses. The questions were reviewed by recognized experts. The survey started with questions for identifying the target responses, including age and identity (patient or caregiver of the patient). Respondents younger than 18 years were forced to end the survey and instructed to pass the survey to their parents or legal guardians. Each participant was automatically directed to either the self-reporting (filled by the patient) or proxy-reporting (filled by the caregiver) version based on their choice of self-identity. The research team provided real-time online support to rare disease patient organizations (RDPOs) during the survey period. Collected answers were manually checked by researchers, with extreme and unusual answers manually verified. The details of questionnaire design, distribution, and answer verification are described in Multimedia Appendix 1.

The questionnaire collected the following information that was reported by the participants or their caregivers: (1) sociodemographic background information (eg, age, sex, educational attainment, and income), (2) diagnosis and treatment (eg, symptom onset and diagnosis, and current treatment status), (3) cost of illness (eg, expenses on medical treatment, traveling, and caretaking), and (4) health-related quality of life (HRQoL).

### Ethical Considerations

Ethical approval was obtained from the Survey and Behavioural Research Ethics Committee of the Chinese University of Hong Kong and the Ethical Committee of Peking Union Medical College Hospital (reference number: SBRE-18-268 and SK-814). All participants were required to read the consent form and click on “consent to participate” before entering the study. All data were anonymized to protect participant privacy and confidentiality. No compensation was provided for participation.

### Participants

Due to the inherent difficulty in identifying and accessing RD populations, nonprobability sampling was used. In 2018, China released the First National List of Rare Diseases, which included 121 different conditions [34]. By 2019, there were established national RDPOs for 56 of these 121 conditions. After inviting all available RDPOs to join the study, 32 RDPOs representing 33 diseases agreed to participate. Participants of this study were recruited through the 32 national RDPOs that represented patients with 33 RDs from China’s first official list of RDs.

Participants meeting the following criteria were considered eligible to join the study: (1) adult patients with RDs who were able to understand and complete the questionnaire and (2) primary caregivers of patients with RDs who were not able to complete the questionnaire owing to limited physical or cognitive function, or age younger than 18 years. A total of 20,804 patients or their caregivers were included.

In this analysis, responses were excluded if (1) the report was completed by proxies of patients who were deceased by the time of the survey (n=18), (2) the patient was younger than 2 years by the time of the survey (n=2122), (3) the respondent did not report detailed information on age, individual income, or out-of-pocket medical expenses of the patient (n=3837). We did not include infants younger than 2 years considering that infants are highly dependent on caretakers and likely demonstrate a unique disease profile compared with children aged older than 2 years. Finally, a total of 16,945 patients or caregivers were included in the analysis. The details of the reasons for exclusion are described in Multimedia Appendix 2.

### Outcome Variables

#### Physical and Mental Health Burden

To allow for comparison with other populations and between different diseases, the perceived general health of patients was evaluated by generic HRQoL instruments. For adult patients, HRQoL was measured by the self-administered 12-item Short-Form Health Survey version 2 (SF-12v2) [35]. The physical component summary score is composed of 4 subscales: Physical Function, Role Physical, Bodily Pain, and General Health, and the mental component summary score is composed of 4 subscales: Mental Health, Role Emotion, Social Function, and Vitality [35]. The response options have 3 levels for Physical Function and 5 levels for the other subscales. In this study, the patient was considered to be presenting “some or severe problems” for the items if the response was “fair/poor” in the General Health subscale, “limited a little/limited a lot” in the Physical Function subscale, “moderately/quite a bit/extremely” in the Bodily Pain subscale, “some of the time/a little of the time/none of the time” in the Mental Health and Vitality subscales, and “all of the time/most of the time/some of the time” in the remaining subscales (Multimedia Appendix 3).

The HRQoL of pediatric patients aged younger than 18 years was measured by PedsQL 4.0 Generic Core Scales, which have been designed for healthy children as well as children with a wide range of health conditions [36]. PedsQL Scales for children aged 2-4, 5-7, 8-12, and 13-18 years measure physical, emotional, social, and school functioning, and have shown good validity and reliability [37]. In this study, the parent-proxy version was completed for all patients aged younger than 18 years. Physical functioning is measured by 8 items, emotional functioning by 5 items, and social functioning by 5 items, with 5-level response options of “never/almost never/sometimes/often/almost always.” In this study, we measured the child’s physical health by physical functioning items and mental health by emotional and social functioning items. The patient was considered to be presenting “some or severe problems” for the items if the response was “sometimes/often/almost always.”

#### Economic Burden

To measure the financial strain borne by the affected individuals and families, this study adopted a patient/individual perspective when evaluating the economic burden. We used proportions of out-of-pocket direct medical, direct nonmedical, and indirect costs divided by the annual household income of patients as indicators to reflect the economic burden on patients and their families. Household income data were collected, including income from production, wage income of household members, transfer income like remittances and welfare, and property income like rent and interest. Out-of-pocket direct medical, direct nonmedical, and indirect costs during the year 2018 were self-reported by the participants.

The questionnaire first asked the participants the range of out-of-pocket expenses on medical treatment in the last year, excluding insurance claims and donations, and then the specific amount within that range. Direct medical costs were measured by out-of-pocket expenses involving the following: (1) medication, including prescribed medication and traditional Chinese medicine; (2) consultation, outpatient care, inpatient care, and surgery, excluding medication; (3) other medical services, such as rehabilitation and physical therapy; and (4) formal care. Direct nonmedical costs were measured by expenses involving the following: (1) traveling related to diagnosis and treatment, including transportation and accommodation, and (2) assistive devices, nutritional supplements, sanitation products, or disability-related home remodeling or renovation. For indirect costs, the income loss of the patient was estimated as follows: (1) full-time employed patients were asked about monthly personal income and lost workdays due to the condition (eg, sick leave and absenteeism) in 2018, and income loss was calculated (lost workdays / total workdays × monthly income × 12) and (2) part-time or self-employed patients, including farmers, were asked to report an estimated annual income loss due to the condition. Additionally, all participants were asked about the estimated annual income loss of caregivers from accompanying the patient. If participants reported no income loss in a component, the cost was considered 0 for that component.

The components of the 3 cost categories are as follows:

- Direct medical costs (out-of-pocket) = medication (eg, prescribed medication and traditional Chinese medicine) + consultation + outpatient care + inpatient care (eg, surgery and chemotherapy) + other medical services (eg, rehabilitation and physical therapy) + formal care
- Direct nonmedical costs = expenses of traveling (eg, transportation and accommodation) + assistive devices + nutritional supplements + sanitation products + disability-related home remodeling or renovation
- Indirect costs = income loss of the patient + income loss of the accompanying persons due to the condition

### Sociodemographic and Disease-Related Factors

Patients’ sociodemographic background information was obtained, including age, sex, educational attainment for adults (below high school, or high school or above), parental educational attainment for children (at least one parent below high school or both parents high school or above), annual household income, and household registration (rural, urban, or overseas). Disease-related information included the time of disease onset evaluated by the time of the first symptom, the time of diagnosis, the time of the most recent treatment, and comorbidities (none or at least one). We calculated household income per person by annual household income divided by the number of household members. This number was then compared with the international poverty line, determined based on the latest criteria set by the World Bank of US $2.15 per person per day [38], which equals to CNY 5380.1 per person per year (US $1=CNY 6.85). Those who fell below the converted poverty line were considered to be in poverty. Active treatment was defined as the most recent treatment within the last 6 months. Delay in diagnosis was defined as the number of years between the onset of symptoms and a definite diagnosis. Disease duration was defined as the number of years between diagnosis and the time of the survey. Recent diagnosis was defined as diagnosis within 1 year before the time of the survey.

### Statistical Analysis

We used cluster analysis to explore the potential homogeneous pattern among patients with RDs according to the physical and mental health burden (adult patients, SF-12 [12 items]; pediatric patients, PedsQL [18 items]), and economic burden (direct medical, direct nonmedical, and indirect costs relative to income) of the disease. The K-prototype algorithm was used for clustering given the mixture of categorical and continuous variables in our data set [39]. The technique aims to group similar subjects together by minimizing the difference between subjects within a group and maximizing the distance between the group centers. This method was exploratory as these indicators have not been previously explored and we had no preconceived ideas about how the patients would cluster. The initial cluster (*k*) values were determined to range from 2 to 6. Two methods were used to determine the optimal number of clusters (*k*): (1) the elbow method, optimal *k* defined by the most significant bend in the graph produced by the elbow technique in the line plot, and (2) the silhouette coefficient, optimal *k* defined as when the coefficient reaches its maximum [40]. The validity of group resolution with optimal *k* was explored by comparing subgroup membership across sociodemographic and disease-related factors using ANOVA, the chi-square test, or the Fisher exact test, as appropriate. The above analyses were conducted using R (Version 4.3.1; R Project for Statistical Computing).

Sociodemographic and disease-related characteristics were described by clusters. The levels of physical and mental health burden, and economic burden were also described. Categorical variables have been presented as number and percentage, and continuous variables have been presented as mean (SD). Differences between each pair of clusters were compared by using pairwise comparison with the Tukey-Kramer adjustment approach. Multinomial regression was conducted to identify sociodemographic and disease-related factors associated with being a member of a cluster. Explanatory variables included age group, sex, educational attainment for adults or parental educational attainment for children, household registration, poverty, active treatment, diagnostic delay, comorbidity, disease duration, and recent diagnosis. Univariable multinomial regression was first conducted, and variables significant at *P*<.10 were further included in multivariable regression to identify the significant factors associated with cluster membership. The significance level was set at *P*<.05. Finally, to obtain an overview of grouping within each specific disease, we also described the distribution of clusters by disease. The above analyses were conducted using Stata 16.0 (Stata Corp).

## Results

### Basic Characteristics

A total of 8454 adult patients and 8491 pediatric patients were included in this analysis. The sociodemographic characteristics of all participants are described in Multimedia Appendix 4. The numbers of adult and pediatric patients by 33 specific diseases according to the International Classification of Diseases and Related Health Problems 10th revision (ICD-10) are described in Multimedia Appendix 5. Among adult patients, the mean age was 37.3 (SD 12.4) years, and 52.9% (4475/8454) of patients were female. The mean disease duration was 9.1 (SD 9.5) years. As described in Multimedia Appendix 6, the proportion of patients reporting “some to severe problems” ranged from 65.2% (5512/8454) to 73.5% (6216/8454) for physical health items and 51.0% (4311/8454) to 74.8% (6319/8454) for mental health items. The mean proportions of annual direct medical, direct nonmedical, and indirect costs relative to annual household income were 105.12%, 40.82%, and 20.98%, respectively. Among pediatric patients, the mean age was 6.7 (SD 3.9) years, and 35.1% (2984/8491) of patients were female. The mean disease duration was 4.6 (SD 3.4) years. The proportion of patients reporting “some to severe problems” ranged from 39.8% (3383/8491) to 67.3% (5716/8491) for physical health items and 46.4% (3943/8491) to 71.4% (6061/8491) for mental health items. As described in Multimedia Appendix 6, the mean proportions of annual direct medical, direct nonmedical, and indirect costs relative to annual household income were 103.22%, 45.30%, and 22.86%, respectively. The scores for each relevant HRQoL item and the amount of disease-related expenses are described in Multimedia Appendix 7.

### Cluster Analysis Results

Results from cluster analysis suggested that a 3-cluster resolution was the best fit for data for both adult and pediatric patients as it showed the most significant bend in the graphs produced by the elbow technique (Multimedia Appendix 8) and the maximum value of silhouette coefficients (Multimedia Appendix 9).

**Figure 1:**
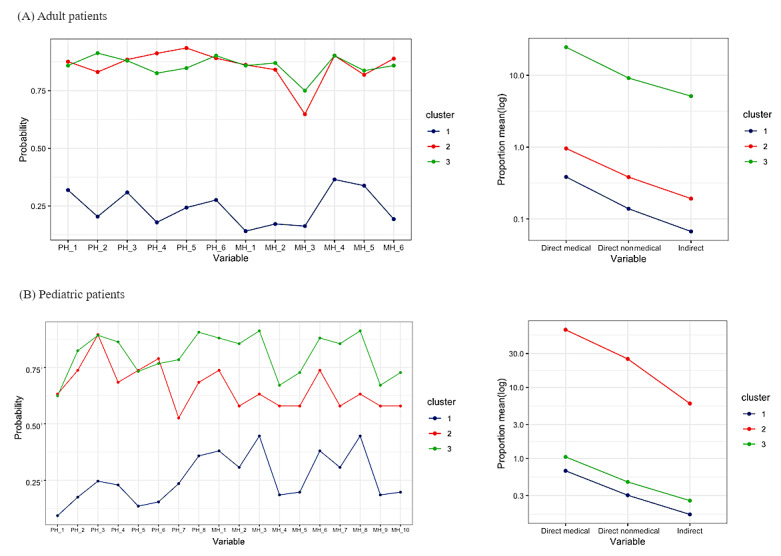
Probability of reporting some or severe problems of physical and mental health items and the mean proportion of out-of-pocket expense categories relative to annual household income by clusters: (A) adult patients; (B) pediatric patients.

The 3 clusters for adult patients are displayed in Figure 1A and described in Multimedia Appendix 6. The first cluster, which has been termed “extremely high burden” (92/8454, 1.1%), was characterized by an elevated likelihood of individuals reporting moderate to severe issues pertaining to both physical and mental health, concomitant with markedly high average levels of out-of-pocket health care expenditure across direct medical, direct nonmedical, and indirect costs relative to household income. The second cluster, which has been termed “overall high burden” (5933/8454, 70.2%), displayed a pronounced probability for participants to report moderate to severe health problems along with high average financial expenditures for diseases relative to household income. Lastly, the third cluster, which has been termed “overall low burden” (2429/8454, 28.7%), represented a lower probability of moderate to severe health-related problems and concurrently low average costs relative to household income.

Similar patterns were observed among pediatric patients, as described in Figure 1B and Multimedia Appendix 6. Consistent with the clusters identified among adult patients, the 3 groups were termed “extremely high burden” (19/8491, 0.2%), “overall high burden” (4864/8491, 57.3%), and “overall low burden” (3608/8491, 42.5%).

### Factors Associated With Cluster Membership

The background characteristics of patients in different clusters are described in Table 1. All variables differed significantly by cluster, except for sex among adult patients and disease duration among pediatric patients in overall and pairwise comparisons. The results of multinomial logistic regression are shown in Table 2. Among adult patients, older age (odds ratio [OR] 1.31, 95% CI 1.17-1.47 for 30-49 years; OR 2.14, 95% CI 1.81-2.53 for 50-69 years; OR 2.29, 95% CI 1.37-3.84 for ≥70 years), lower education (OR 2.16, 95% CI 1.91-2.43), poverty (OR 3.31, 95% CI 2.70-4.07), active treatment (OR 1.24, 95% CI 1.11-1.37), diagnostic delay (OR 1.34, 95% CI 1.16-1.55 for 1-2 years; OR 1.36, 95% CI 1.18-1.58 for ≥3 years), comorbidity (OR 2.45, 95% CI 2.21-2.72), and longer disease duration (OR 1.01, 95% CI 1.00-1.02 for each 1-year increase) predicted membership in the overall high burden group compared with the overall low burden group. Similar predictors were found to be associated with the group having extremely high burden: lower education (OR 2.57, 95% CI 1.62-4.09), poverty (OR 50.37, 95% CI 29.40-86.30), 1-2 years of diagnostic delay (OR 2.04, 95% CI 1.17-3.56), and comorbidity (OR 4.57, 95% CI 2.67-7.84).

**Table 1:** Demographic and disease-related characteristics in patient subgroups among adult and pediatric patients.

Variable		Adult patients (n=8454)					Pediatric patients (n=8491)			
		Overall low (n=2429)	Overall high (n=5933)	Extremely high (n=92)	*P* value^a^	Overall low (n=3608)		Overall high (n=4864)	Extremely high (n=19)	*P* value^b^
Age (years), mean (SD)		34.32 (11.18)^c^	38.52 (12.71)^d^	35.43 (10.92)	<.001	5.96 (3.63)^c^		7.22 (3.96)	8.00 (4.36)	<.001
**Sex, n (%)**					.06					<.001
	Male	1102 (45.4)	2827 (47.6)	50 (54.3)		2105 (58.3)^c^		3390 (69.7)	12 (63.2)	
	Female	1327 (54.6)	3106 (52.4)	42 (45.7)		1503 (41.7)^c^		1474 (30.3)	7 (36.8)	
**Educational attainment^e^, n (%)**					<.001					<.001
	Below high school	486 (20.0)^c,d^	2320 (39.1)^d^	52 (56.5)		1518 (42.2)^c,d^		2636 (54.5)^d^	15 (83.3)	
	High school or above	1943 (80.0)^c,d^	3613 (60.9)^d^	40 (43.5)		2075 (57.8)^c,d^		2203 (45.5)^d^	3 (16.7)	
**Household registration, n (%)**					<.001					<.001
	Rural	946 (38.9)^c,d^	2772 (46.7)^d^	60 (65.2)		1875 (52.0)^c^		2839 (58.4)	13 (68.4)	
	Urban or overseas	1483 (61.1)^c,d^	3161 (53.3)^d^	32 (34.8)		1733 (48.0)^c^		2025 (41.6)	6 (31.6)	
**Poverty, n (%)**					<.001					<.001
	Nonpoverty	2302 (94.8)^c,d^	4934 (83.2)^d^	22 (23.9)		3129 (86.7)^c,d^		4020 (82.6)^d^	3 (15.8)	
	Poverty	127 (5.2)^c,d^	999 (16.8)^d^	70 (76.1)		479 (13.3)^c,d^		844 (17.4)^d^	16 (84.2)	
**Active treatment, n (%)**					.007					<.001
	Nonactive	963 (39.6)^c^	2137 (36.0)	32 (34.8)		679 (18.8)^c^		1468 (30.2)	4 (21.1)	
	Active	1466 (60.4)^c^	3796 (64.0)	60 (65.2)		2929 (81.2)^c^		3396 (69.8)	15 (78.9)	
**Delay in diagnosis, n (%)**					<.001					<.001
	<1 year	1725 (72.5)^c^	3865 (66.5)	58 (64.4)		3341 (93.3)^c^		3925 (81.3)	15 (83.3)	
	1-2 years	330 (13.9)^c^	959 (16.5)	19 (21.1)		190 (5.3)^c^		669 (13.9)	3 (16.7)	
	≥3 years	324 (13.6)^c^	988 (17.0)	13 (14.4)		51 (1.4)^c^		234 (4.8)	0 (0)	
**Comorbidity, n (%)**					<.001					<.001
	None	1273 (52.4)^c,d^	1734 (29.2)	19 (20.7)		2850 (79.0)^c^		2512 (51.6)	10 (52.6)	
	At least one	1155 (47.6)^c,d^	4198 (70.8)	73 (79.3)		758 (21.0)^c^		2352 (48.4)	9 (47.4)	
Disease duration (years), mean (SD)		8.29 (8.90)^c^	9.38 (9.68)	8.40 (9.07)	<.001	4.57 (3.34)		4.63 (3.50)	6.05 (4.65)	.12
**Recent diagnosis, n (%)**					.006					<.001
	No	1925 (79.3)^c^	4877 (82.2)	73 (79.3)		3151 (87.3)^c^		4014 (82.5)	15 (78.9)	
	Yes	504 (20.7)^c^	1056 (17.8)	19 (20.7)		457 (12.7)^c^		850 (17.5)	4 (21.1)	

^a^*P* value of ANOVA for continuous variables; chi-square test for other categorical variables.

^b^*P* value of ANOVA for continuous variables; chi-square test for sex, household registration, and comorbidity; Fisher exact test for other categorical variables.

^c^Significantly different from the overall high cluster at the *P*<.05 level.

^d^Significantly different from the extremely high cluster at the *P*<.05 level.

^e^Parental education (at least one parent below high school or both parents high school or above) for pediatric patients.

**Table 2:** Multivariable multinomial logistic regression of cluster membership as predicted by sociodemographic and disease-related factors according to adult and pediatric patients.

Variable		Adult patients^a^					Pediatric patients^a^			
		Cluster 2: overall high, OR^b^ (95% CI)	*P* value	Cluster 3: extremely high, OR (95% CI)	*P* value	Cluster 2: overall high, OR (95% CI)		*P* value	Cluster 3: extremely high, OR (95% CI)	*P* value
**Sex**					<.001					<.001
	Male	—^c^		—		Reference			Reference	
	Female	—		—		0.63 (0.57-0.70)		<.001	0.56 (0.20-1.63)	.29
**Age (years)^d^**										
	18-29/2-5	Reference		Reference		Reference			Reference	
	30-49/6-12	1.31 (1.17-1.47)	<.001	0.90 (0.55-1.44)	.65	1.79 (1.58-2.02)		<.001	1.15 (0.30-4.45)	.84
	50-69/13-17	2.14 (1.81-2.53)	<.001	1.05 (0.5-2.23)	.89	2.58 (2.04-3.26)		<.001	0.91 (0.08-10.13)	.94
	≥70/N/A^e^	2.29 (1.37-3.84)	.002	N/A	.99					
**Education/parental education^f^**										
	High school or above	Reference		Reference		Reference			Reference	
	Below high school	2.16 (1.91-2.43)	<.001	2.57 (1.62-4.09)	<.001	1.46 (1.32-1.61)		<.001	1.94 (0.51-7.40)	.33
**Poverty**										
	Nonpoverty	Reference		Reference		Reference			Reference	
	Poverty	3.31 (2.70-4.07)	<.001	50.37 (29.40-86.30)	<.001	1.14 (0.99-1.31)		.06	22.83 (6.08-85.79)	<.001
**Active treatment**										
	Nonactive	Reference		Reference		Reference			Reference	
	Active	1.24 (1.11-1.37)	<.001	1.44 (0.91-2.28)	.12	0.57 (0.51-0.63)		<.001	0.85 (0.27-2.66)	.78
**Delay in diagnosis**										
	<1 year	Reference		Reference		Reference			Reference	
	1-2 years	1.34 (1.16-1.55)	<.001	2.04 (1.17-3.56)	.01	2.50 (2.09-2.98)		<.001	3.50 (0.95-12.92)	.06
	≥3 years	1.36 (1.18-1.58)	<.001	1.22 (0.65-2.30)	.54	2.10 (1.51-2.92)		<.001	N/A	N/A
**Comorbidity**										
	None	Reference		Reference		Reference			Reference	
	At least one	2.45 (2.21-2.72)	<.001	4.57 (2.67-7.84)	<.001	3.51 (3.17-3.89)		<.001	2.89 (1.10-7.59)	.03
**Recent diagnosis**										
	No	—		—		Reference			Reference	
	Yes	—		—		1.27 (1.08-1.48)		.004	4.12 (0.81-20.98)	.09
Disease duration^g^		1.01 (1.00-1.02)	.001	0.98 (0.95-1.01)	.12	0.95 (0.93-0.97)		<.001	1.17 (0.94-1.45)	.16

^a^The overall low impact group (cluster 1) is the reference group.

^b^OR: odds ratio.

^c^Not included in the final model due to nonsignificant results.

^d^Age categories: 18-29, 30-49, 50-69, and ≥70 years for adult patients; 2-5, 6-12, and 13-17 years for pediatric patients.

^e^N/A: not applicable.

^f^Parental education (at least one parent below high school and both parents high school or above) for pediatric patients.

^g^Continuous variable.

The predictors identified among pediatric patients were largely similar. Among these patients, older age (OR 1.79, 95% CI 1.58-2.02 for 2-5 years; OR 2.58, 95% CI 2.04-3.26 for 13-17 years), lower parental education (OR 1.46, 95% CI 1.32-1.61), diagnostic delay (OR 2.50, 95% CI 2.09-2.98 for 1-2 years; OR 2.10, 95% CI 1.51-2.92 for ≥3 years), comorbidity (OR 3.51, 95% CI 2.17-3.89), and recent diagnosis (OR 1.27, 95% CI 1.08-1.48) predicted membership in the overall high burden group compared with the overall low burden group. Conversely, male sex (OR 0.63, 95% CI 0.57-0.70), not being under active treatment (OR 0.57, 95% CI 0.51-0.63), and shorter disease duration (OR 0.95, 95% CI 0.93-0.97 for each 1-year increase) were associated with a lower likelihood of being in the overall high burden group than in the low burden group. In the extremely high burden group, the only significant predictors were poverty (OR 22.83, 95% CI 6.08-85.79) and comorbidity (OR 2.89, 95% CI 1.10-7.59).

### Cluster Distribution by Disease

**Figure 2:**
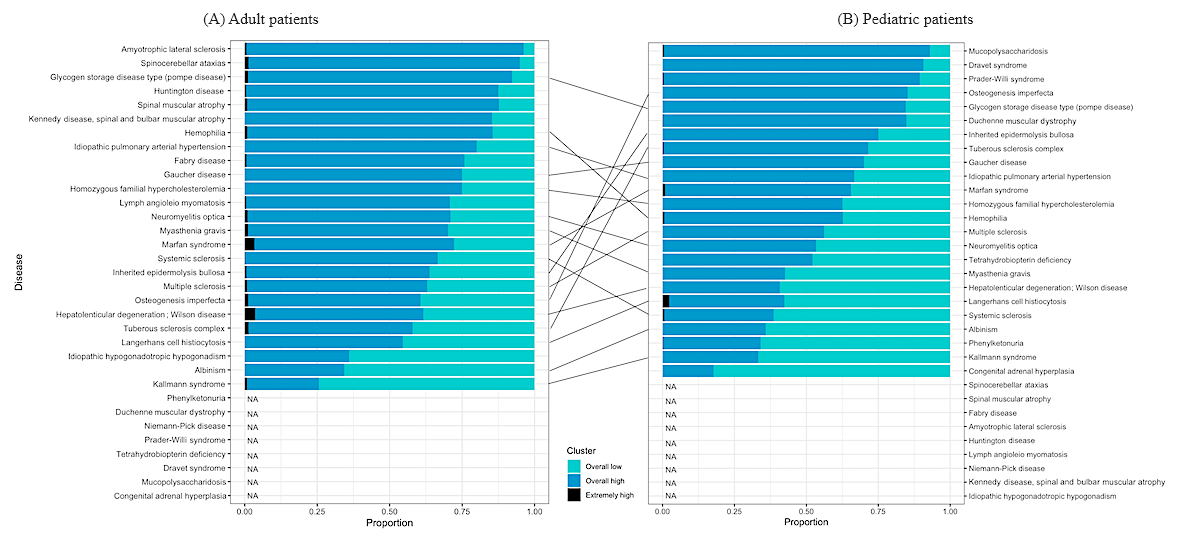
Proportion of clusters by disease: (A) adult patients; (B) pediatric patients. NA: data not presented as the number of observations was <10%.

Figure 2 describes the proportion of clusters according to different diseases in adult and pediatric patients. To avoid potential bias, the cluster distribution was not presented if the number of adult or pediatric patients constituted less than 10% of the total number of patients with the specific disease. Among adult patients, those with Wilson disease, Marfan syndrome (MFS), spinocerebellar ataxia (SCA), and tuberous sclerosis complex had the highest proportion clustered in the “extremely high burden” group. Those with amyotrophic lateral sclerosis (ALS), SCA, Pompe disease, and Huntington disease (HD) had the highest proportion clustered in the “overall high burden” group. Those with Kallmann syndrome, albinism, and idiopathic hypogonadotropic hypogonadism (IHH) had the highest proportion clustered in the “overall low burden” group.

The distribution of clusters differed among pediatric patients. Patients with Langerhans cell histiocytosis (LCH), MFS, hemophilia, and systemic sclerosis (SSc) were most highly clustered in the “extremely high burden” group among all patients. Those with mucopolysaccharidoses (MPS), Dravet syndrome (DS), Prader-Willi syndrome (PWS), and osteogenesis imperfecta had the highest proportion clustered in the “overall high burden” group. Those with congenital adrenal hyperplasia (CAH), Kallmann syndrome, phenylketonuria, and albinism had the highest proportion clustered in the “overall low burden” group.

## Discussion

### Main Findings

This study identified 3 distinct groups of patients with 33 RDs in China, with similar patterns observed for adult patients and pediatric patients. The first group was characterized by high physical and mental health burden, as well as extremely high economic burden. Adults with Wilson disease, MFS, and SCA and children with LCH, MFS, and SSc tended to cluster in this group. The second group was associated with high physical and mental health burden, and high economic burden. Patients with diseases that affect the nervous system, cause neuromuscular symptoms, and require long-term treatment were in this group (eg, ALS, SCA, and Pompe disease for adults; MPS and DS for children). The third group involved low burden in physical and mental health and economic aspects. Diseases that affect the endocrine system and show symptoms that are controllable under appropriate treatment tended to present in this group (eg, Kallmann syndrome and IHH for adults, and CAH for children). Those who were older, had a lower socioeconomic status (SES), experienced diagnostic delay, and had comorbidity were consistently more likely to demonstrate an overall high burden of RDs. In particular, patients in poverty were significantly more vulnerable to an extremely high burden.

### Clusters and Predictors of Membership

Our results show that among RD patients in China, poor physical and mental health status is clustered with high direct and indirect costs of the disease, which is consistent with previous findings linking poor quality of life (QoL) with high economic costs in people living with acromegaly [41] and hemophilia [42]. Notably, in each cluster, the levels of direct medical, direct nonmedical, and indirect costs showed a consistent pattern. This suggests that the complexity of treatment and diagnosis associated with the condition coincides with increased cross-region health care–seeking behavior as well as lost working hours and undermines productivity among patients and caregivers.

The majority of adult (5933/8454, 70.2%) and pediatric (4864/8491, 58.1%) patients were clustered into the group presenting an overall high burden. Older age and a lower SES were found to significantly predict high overall burden, consistent with previous findings from the United States that RD patients who were older and had lower income experienced poor HRQoL [18]. This may be attributable to the fact that physical function limitations are expected in older age as the disease progresses and that those with a higher SES are more likely to afford access to quality health care and to travel a greater distance to seek medical resources. Comorbidity was unsurprisingly a predictor as it can add to physical symptoms and more complex use of medications. Our finding regarding the association between delayed diagnosis and high overall burden indirectly supports the notion that longer diagnostic delay may lead to a greater symptom burden at the time of initial diagnosis, which subsequently affects the health outcomes and increases the associated direct and indirect costs [43]. Very small proportions of adult (92/8454, 1.1%) and pediatric (19/8491, 0.2%) patients were clustered into the group with extremely high burden. SES, particularly poverty, was a significant predictor of the membership of this cluster among both adult and pediatric patients. This corroborates the results from other regions that lower income is associated with a higher financial burden of RDs [44,45]. A study from China reported that 84.2% and 45.9% of families with pediatric and adult patients with Pompe disease, respectively, were forced to live in poverty due to illness [28]. These findings together indicate that RDs impose significant financial burdens that tend to disproportionately affect individuals who are already socioeconomically disadvantaged, and the diagnosis of an RD may be catastrophic to a household in poverty.

A few factors showed distinct associations between adult and pediatric patients. Male sex was only significant in predicting overall high burden among pediatric patients, which may be related to disproportionate disease severity and gender inequality in resource utilization influenced by sociocultural norms. Previously, disease duration was found to be associated with poorer mental health in adult RD patients in Italy [46] but with higher QoL among adults in the United States [18]. In our study, longer duration of disease and active treatment were identified as risk factors of an overall high burden among adults but protective factors among children. This finding may indicate the following: (1) as the disease progresses in adults, the symptom burden and medical expenses tend to accumulate, resulting in increased overall burden; (2) children are more likely to receive appropriate timely treatment and support after diagnosis, which can help alleviate their health burden; (3) adult patients commonly bear the financial burden associated with their illness, which may affect their mental health and choice of treatment, whereas such a burden for pediatric patients is typically borne by their families. Poverty significantly predicted an overall high burden only among adults, possibly because adults in China tend to avoid using health care to a certain degree to save money for the family but would willingly spend more in an attempt to save their children.

### Cluster Distribution by Disease

Among patients with 33 RDs, we found that adults with ALS, SCA, and Pompe disease and children with MPS, DS, and PWS were prone to overall high burdens in health and economic aspects. These diseases are either neurologic or metabolic, causing severe impacts on muscles and progressive impairments in physical functioning. This is consistent with the conclusion of a meta-analysis on diverse RDs, which indicated that patients with musculoskeletal diseases showed the lowest QoL [47]. Pompe disease and MPS can be treated with enzyme replacement therapy, which is extremely costly, with only a small proportion covered by social or commercial insurance schemes in China [28]. The high out-of-pocket medical expenses may thereby contribute to the underdosed use of drugs and suboptimal management of symptoms. There are no disease-modifying therapies for ALS, SCA, HD, DS, and PWS, but patients usually undergo long-term treatments to slow symptom progression and prevent complications. These conditions are generally characterized by severe symptoms and complications without effective or affordable treatments, and failure to receive treatment in turn adds to the patient’s health burden.

Adults with Wilson disease, MFS, and SCA, and children with LCH, MFS, and hemophilia were more likely to experience a high burden, particularly economically, than those with other conditions, although the absolute number of patients in this group was low. These diseases consistently cause muscular symptoms, which can lead to physical dysfunctions. Wilson disease, MFS, and SCA are all progressive in nature, affect multiple organ systems, and often require complex and specialized treatments, including surgeries, medications, and prolonged therapies. For example, Wilson disease and hemophilia often require long-term pharmaceutical treatment, and MFS often needs regular cardiovascular monitoring. The treatments can also include emergency services and hospitalization for LCH; surgery for MFS, Wilson disease, and LCH; and rehabilitation for SCA. These diseases, unlike others that tend to cause an overall high burden, often have available but costly therapies. The need for complex prolonged treatments and often life-long management further exacerbates the economic burden on patients and their families.

Adults with Kallmann syndrome, albinism, and IHH, and children with CAH, Kallmann syndrome, and phenylketonuria more likely demonstrated an overall low burden. All these conditions are classified under the ICD-10 category of “endocrine, nutritional, and metabolic diseases,” and they are generally nonprogressive, with relatively moderate symptoms that are preventable or manageable by appropriate strategies. Hormone therapy, commonly used for endocrine disorders, is covered by medical insurance in China. Although these conditions present a comparatively lower burden than the other RDs examined, it should be noted that patients still face the challenges of life-long management and social integration.

### Implications

The distinct clusters identified in this study highlight a need for health care interventions that are tailored to the specific burden experienced by different patient groups. For adult and pediatric patients with overall high burden and potentially high unmet needs, health care services need to integrate psychological support with routine care to address the significant physical and mental health burden. Mental and social support programs, such as psychological counseling and social integration initiatives, can help patients and their families cope with daily challenges. To relieve the economic burden, policy makers should consider adopting financial support mechanisms, such as subsidies, improved insurance coverage, and financial aid programs, especially for treatments like enzyme replacement therapy that incur high out-of-pocket costs. As patients often seek cross-region health care in regions like China where medical resources are unevenly distributed, providing subsidies for traveling may help relieve the financial burden on patients and reduce barriers to health care access. Considering the potential impact of RDs on patients’ ability to work, support measures, such as workplace accommodations and public awareness campaigns, may be beneficial to address the indirect costs for the RD population.

The finding that diagnostic delay contributes to high overall burden indicates that receiving a correct and timely diagnosis can alleviate the burden of having an RD. To minimize diagnostic delay, it is imperative to encourage investment in the development of diagnosis techniques, improve expertise and knowledge related to the diagnosis of RDs among health care providers, and enhance the referral system. The significant association between lower SES and higher disease burden underscores an urgent need to promote equitable health care access. Potential policy actions include sliding scale payment options and ensuring the availability of health care facilities in lower-income areas. Additional financial assistance can be provided to socioeconomically disadvantaged households to prevent further impoverishment related to the illness. The divergent association for active treatment between adult and pediatric patients suggests that it may be beneficial to promote patient-centered care for adults and to include the families of pediatric patients in psychosocial support services as they experience caretaking and financial burdens.

Our findings also shed some light on which diseases could be prioritized when allocating resources in policy-making. Diseases identified to be associated with an overall high burden likely present high unmet needs for patients. Those without effective treatment, such as ALS, SCA, MPS, and DS, should be prioritized in initiatives that encourage innovation for treatment as well as in social protection policies. Those with effective but costly treatment, such as Pompe disease, should be prioritized in national health insurance schemes to improve the availability and accessibility of therapies. Continuous efforts are needed to facilitate the inclusion of more drugs in insurance schemes and expedite the process of repurposing existing drugs. Patients with diseases, such as Wilson disease, MFS, LCH, and hemophilia, and those with a lower SES should be provided with financial support, such as subsidies and charity, to relieve the burden from long-term costly treatment. For diseases with relatively moderate symptoms and a lower economic burden, such as Kallmann syndrome, albinism, and CAH, the priority strategy can involve providing informational, psychological, and social support of disease management for patients and their families.

### Strengths and Limitations

This study has provided valuable evidence on the burden of RDs by incorporating indicators of physical and mental well-being as well as the direct medical, nonmedical, and indirect costs of the disease. With the increasing research interest to consider diverse RDs as a collective disease, this study provides a novel approach to classify RDs by their health and economic burden compared with the traditional classification according to affected systems. The findings have generated implications for the management of RD patients in China and other developing settings at the preliminary stage of implementing RD policies. Other strengths of this study include the large sample size, which increased the validity of the results, and the stratified analysis by adult and pediatric populations, which identified their unique challenges.

There are some limitations in this study. First, the data collected were self-reported and may involve reporting bias. Second, different HRQoL instruments were used to measure the HRQoL of adult and pediatric patients, which precludes the direct comparison of health burden between the 2 populations. However, adopting a pediatric-specific instrument produces more accurate results for children than applying generic instruments designed for adults. Third, the causal relationship between sociodemographic and clinical factors and cluster membership cannot be established due to the cross-sectional design of the study. Fourth, due to the lack of a complete sampling frame of RD patients in China, we used a nonprobability approach to recruit patients through RDPOs. The current sampling and internet-based data collection approaches, although expedient in the RD context of China, may inevitably introduce selection bias as they tend to exclude individuals from rural or lower socioeconomic backgrounds who cannot access the internet or RDPOs. Nonetheless, as China has a large number of people living with RDs compared to other regions [48], this study provides valuable data from a large nationwide sample. Lastly, the study used HRQoL items to reflect the health burden of disease and adopted a patient/individual perspective when evaluating the economic burden. While such an approach provides important insights for patient-centered interventions and support systems, future studies may consider measuring the burden more comprehensively by adopting a health care perspective or including a wider variety of indicators. To our knowledge, this study is the first to explore the underlying patterns of the health and economic burden of RDs. More evidence from other regions is needed to validate our results.

### Conclusions

Heavy health burden tends to cluster with high economic burden in adult and pediatric RD populations in China. Older age, lower SES, diagnostic delay, and comorbidity contribute to high health and economic burden among patients with RDs. Patients with diseases that show high severity and require long-term costly treatment are prone to high overall burden. Health and social policies and interventions, such as investment in RD diagnostic techniques, psychological counseling, social integration initiatives, and improved insurance coverage, are needed to improve the accessibility of RD treatment and alleviate burden among patients and their families.

## Appendix

Multimedia Appendix 1 Details of questionnaire design, survey distribution, and answer verification.

Multimedia Appendix 2 Selection of participants included in the analysis.

Multimedia Appendix 3 Short-Form Health Survey items and responses indicating some or severe problems.

Multimedia Appendix 4 Demographic and disease-related characteristics among all participants (n=16,945).

Multimedia Appendix 5 Number of participants according to disease and ICD-10 codes.

Multimedia Appendix 6 Probability of reporting some or severe problems of physical and mental health items and the mean proportion of out-of-pocket expense categories relative to annual household income by clusters.

Multimedia Appendix 7 Quality of life instrument scores and annual disease-related expenses (in US$) by clusters.

Multimedia Appendix 8 Number of clusters determined with the elbow method: (A) adult patients; (B) pediatric patients.

Multimedia Appendix 9 Silhouette coefficients and number of clusters by adult and pediatric patients.

## Abbreviations

ALS: amyotrophic lateral sclerosis
CAH: congenital adrenal hyperplasia
DS: Dravet syndrome
HD: Huntington disease
HRQoL: health-related quality of life
ICD-10: International Classification of Diseases and Related Health Problems 10th revision
IHH: idiopathic hypogonadotropic hypogonadism
LCH: Langerhans cell histiocytosis
MFS: Marfan syndrome
MPS: mucopolysaccharidoses
OR: odds ratio
PWS: Prader-Willi syndrome
RD: rare disease
RDPO: rare disease patient organization
SCA: spinocerebellar ataxia
SES: socioeconomic status
SF-12v2: 12-item Short-Form Health Survey version 2
SSc: systemic sclerosis
UN: United Nations

## Data Availability

Derived data supporting the findings of this study are available from the corresponding author upon reasonable request.

## References

[1] Richter T, Nestler-Parr S, Babela R, Khan ZM, Tesoro T, Molsen E, Hughes DA, International Society for Pharmacoeconomics and Outcomes Research Rare Disease Special Interest Group (2015). Rare disease terminology and definitions-a systematic global review: report of the ISPOR Rare Disease Special Interest Group. Value Health.

[2] Field MJ, Boat TF, Institute of Medicine (US) Committee on Accelerating Rare Diseases Research and Orphan Product Development (2010). Rare Diseases and Orphan Products: Accelerating Research and Development.

[3] Improving access to orphan medicines for all affected EU citizens. European Commission.

[4] Rare Diseases at FDA. US FDA.

[5] Nguengang Wakap S, Lambert DM, Olry A, Rodwell C, Gueydan C, Lanneau V, Murphy D, Le Cam Y, Rath A (2020). Estimating cumulative point prevalence of rare diseases: analysis of the Orphanet database. Eur J Hum Genet.

[6] Angelis A, Tordrup D, Kanavos P (2015). Socio-economic burden of rare diseases: A systematic review of cost of illness evidence. Health Policy.

[7] Limb L, Nutt S, Sen A (2010). Experiences of Rare Diseases: An Insight from Patients and Families.

[8] Aymé S, Kole A, Groft S (2008). Empowerment of patients: lessons from the rare diseases community. Lancet.

[9] Schieppati A, Henter J, Daina E, Aperia A (2008). Why rare diseases are an important medical and social issue. The Lancet.

[10] Yang G, Cintina I, Pariser A, Oehrlein E, Sullivan J, Kennedy A (2022). The national economic burden of rare disease in the United States in 2019. Orphanet J Rare Dis.

[11] Addressing the challenges of persons living with a rare disease and their families. United Nations.

[12] Navarrete-Opazo AA, Singh M, Tisdale A, Cutillo CM, Garrison SR (2021). Can you hear us now? The impact of health-care utilization by rare disease patients in the United States. Genet Med.

[13] Walker CE, Mahede T, Davis G, Miller LJ, Girschik J, Brameld K, Sun W, Rath A, Aymé S, Zubrick SR, Baynam GS, Molster C, Dawkins HJ, Weeramanthri TS (2017). The collective impact of rare diseases in Western Australia: an estimate using a population-based cohort. Genet Med.

[14] Chiu ATG, Chung CCY, Wong WHS, Lee SL, Chung BHY (2018). Healthcare burden of rare diseases in Hong Kong - adopting ORPHAcodes in ICD-10 based healthcare administrative datasets. Orphanet J Rare Dis.

[15] Cai X, Yang H, Genchev GZ, Lu H, Yu G (2019). Analysis of economic burden and its associated factors of twenty-three rare diseases in Shanghai. Orphanet J Rare Dis.

[16] Divino V, DeKoven M, Kleinrock M, Wade RL, Kim T, Kaura S (2016). Pharmaceutical expenditure on drugs for rare diseases in Canada: a historical (2007-13) and prospective (2014-18) MIDAS sales data analysis. Orphanet J Rare Dis.

[17] Chung CC, Ng NY, Ng YN, Lui AC, Fung JL, Chan MC, Wong WH, Lee SL, Knapp M, Chung BH (2023). Socio-economic costs of rare diseases and the risk of financial hardship: a cross-sectional study. Lancet Reg Health West Pac.

[18] Bogart KR, Irvin VL (2017). Health-related quality of life among adults with diverse rare disorders. Orphanet J Rare Dis.

[19] Ng YN, Ng NY, Fung JL, Lui AC, Cheung NY, Wong WH, Lee SL, Knapp M, Chung CC, Chung BH (2022). Evaluating the health-related quality of life of the rare disease population in Hong Kong using EQ-5D 3-level. Value Health.

[20] Xu RH, Ng SSM, Luo N, Dong D, Zhang S (2023). Measurement of health-related quality of life in individuals with rare diseases in China: Nation-wide online survey. JMIR Public Health Surveill.

[21] Bisquera A, Gulliford M, Dodhia H, Ledwaba-Chapman L, Durbaba S, Soley-Bori M, Fox-Rushby J, Ashworth M, Wang Y (2021). Identifying longitudinal clusters of multimorbidity in an urban setting: A population-based cross-sectional study. Lancet Reg Health Eur.

[22] Zhou J, Wei M, Zhang J, Liu H, Wu C (2022). Association of multimorbidity patterns with incident disability and recovery of independence among middle-aged and older adults. Age Ageing.

[23] Li X, Zhang X, Zhang S, Lu Z, Zhang J, Zhou J, Li B, Ou L (2021). Rare disease awareness and perspectives of physicians in China: a questionnaire-based study. Orphanet J Rare Dis.

[24] Ying Z, Gong L, Li C (2021). An update on China's national policies regarding rare diseases. Intractable Rare Dis Res.

[25] Zhao Z, Pei Z, Hu A, Zhang Y, Chen J (2023). Analysis of incentive policies and initiatives on orphan drug development in China: Challenges, reforms and implications. Orphanet J Rare Dis.

[26] Zhao W (2022). China's campaign against rare diseases. Natl Sci Rev.

[27] Huang R, Shao W (2020). China Rare Disease Drug Accessibility Report 2019.

[28] Chen S, Dong D (2023). Improving insurance protection for rare diseases: Economic burden and policy effects - simulation of people with Pompe disease in China. Int J Health Policy Manag.

[29] Mengyuan F (2018). Medical service utilisation, economic burden and health status of patients with rare diseases in China. J Chin Pharm Sci.

[30] Liang T (2016). Social work services for patients with rare diseases: real needs, main difficulties and countermeasures. Soc Work Manag.

[31] Dong D, Chung RY, Chan RHW, Gong S, Xu RH (2020). Why is misdiagnosis more likely among some people with rare diseases than others? Insights from a population-based cross-sectional study in China. Orphanet J Rare Dis.

[32] Kaplowitz MD, Hadlock TD, Levine R (2004). A comparison of web and mail survey response rates. Public Opinion Quarterly.

[33] Milton AC, Ellis LA, Davenport TA, Burns JM, Hickie IB (2017). Comparison of self-reported telephone interviewing and web-based survey responses: Findings from the Second Australian Young and Well National Survey. JMIR Ment Health.

[34] He J, Kang Q, Hu J, Song P, Jin C (2018). China has officially released its first national list of rare diseases. Intractable Rare Dis Res.

[35] Ware J, Kosinski M, Keller S (1996). A 12-Item Short-Form Health Survey: construction of scales and preliminary tests of reliability and validity. Med Care.

[36] Varni J, Seid M, Rode C (1999). The PedsQL: measurement model for the pediatric quality of life inventory. Med Care.

[37] Varni JW, Burwinkle TM, Seid M (2006). The PedsQL 4.0 as a school population health measure: feasibility, reliability, and validity. Qual Life Res.

[38] September 2022 Update to the Poverty and Inequality Platform (PIP): What’s New. World Bank.

[39] Madhuri R, Murty MR, Murthy JVR, Reddy PVGDP, Satapathy SC, Satapathy S, Avadhani P, Udgata S, Lakshminarayana S (2014). Cluster Analysis on Different Data Sets Using K-Modes and K-Prototype Algorithms. ICT and Critical Infrastructure: Proceedings of the 48th Annual Convention of Computer Society of India-Vol II. Advances in Intelligent Systems and Computing, vol 249.

[40] Rousseeuw PJ (1987). Silhouettes: A graphical aid to the interpretation and validation of cluster analysis. Journal of Computational and Applied Mathematics.

[41] Liu S, Adelman DT, Xu Y, Sisco J, Begelman SM, Webb SM, Badia X, Thethi TK, Fonseca V, Shi L (2018). Patient-centered assessment on disease burden, quality of life, and treatment satisfaction associated with acromegaly. J Investig Med.

[42] Kodra Y, Cavazza M, Schieppati A, De Santis M, Armeni P, Arcieri R, Calizzani G, Fattore G, Manzoli L, Mantovani L, Taruscio D (2014). The social burden and quality of life of patients with haemophilia in Italy. Blood Transfus.

[43] Tejwani V, Nowacki AS, Fye E, Sanders C, Stoller JK (2019). The impact of delayed diagnosis of alpha-1 antitrypsin deficiency: The association between diagnostic delay and worsened clinical status. Respir Care.

[44] Koçkaya G, Oguzhan G, Ökçün S, Kurnaz M (2023). Out-of-pocket healthcare expenditures of Turkish households living with rare diseases. Front Public Health.

[45] Juyani Y, Hamedi D, Hosseini Jebeli SS, Qasham M (2016). Multiple sclerosis and catastrophic health expenditure in Iran. Glob J Health Sci.

[46] Pasculli G, Resta F, Guastamacchia E, Di Gennaro L, Suppressa P, Sabbà C (2004). Health-related quality of life in a rare disease: hereditary hemorrhagic telangiectasia (HHT) or Rendu-Osler-Weber disease. Qual Life Res.

[47] Sequeira AR, Mentzakis E, Archangelidi O, Paolucci F (2021). The economic and health impact of rare diseases: A meta-analysis. Health Policy and Technology.

[48] Wang J, Guo JJ, Yang L, Zhang Y, Sun Z, Zhang Y (2010). Rare diseases and legislation in China. The Lancet.

