# Supplementary material for: Patterns of the Health and Economic Burden of 33 Rare Diseases in China: Nationwide Web-Based Study
Source: JMIR Public Health Surveill. 2024 Aug 27;10:e57353. doi: 10.2196/57353 (PMC11387910; doi:10.2196/57353)
Supplement: Multimedia Appendix 1 [file publichealth_v10i1e57353_app1.docx]

**Multimedia Appendix 1.** Details of questionnaire design, survey distribution, and answer verification.

Data collection details

The questionnaire was co-produced by the research team and representatives from the 32 rare disease patient organizations (RDPOs). Questions related to diagnosis, symptoms, treatments, and costs were discussed thoroughly through two rounds of in-person meetings and multiple rounds of online communications. The first three drafts of the questionnaire were reviewed by recognized experts in the field of RD research in China and pilot tested with patients and caregivers recommended by each RDPO. After the questionnaire was finalized, each RDPO distributed the survey link among their own patient community, which requires all community members to have a confirmed diagnosis. For each participant, after they completed the survey, they were provided with a unique QR code that they can share with the RDPO that they were affiliated with. Hence, from the RDPO’s side, the patient organization would know the identity of the patient or caregiver associated with this particular QR code, whereas the answers were anonymous to the research team. Such a design guaranteed the privacy of the patients/caregivers, while making it possible to have verification of data authenticity.

Research team provided real-time online support to the RDPOs during the five months of survey. When the survey ended, answers were manually checked by three research assistants. Extreme values and unusual answers were manually verified by trained staff by confirming with the patient or caregiver. Answers, such as income or expenditure, that did not seem to be usual were sent to the RDPO along with the QR code. So that the RDPO could contact patient/caregiver directly and to verify their answers. If the answer was simply a typo, such as entering one more 0, the research team would manually correct the answers. If the patient/caregiver refused to verify, though occurred very infrequently, the answers would be regarded as “missing” in the final analysis.

List of 32 RDPOs involved in recruitment of participants and data collection

|  | Names in Chinese | Name in English |
| --- | --- | --- |
|  | 北京爱力重症肌无力罕见病关爱中心 | Beijing Aili Myasthenia Gravis Care Center |
|  | 北京爱稀客肺动脉高压罕见病关爱中心 | iSEEK PH Hope Center |
|  | 北京病痛挑战公益基金会多发性硬化项目组 | The Illness Challenge Foundation Multiple Sclerosis Project Group |
|  | 北京东方丝雨渐冻人罕见病关爱中心 | Oriental Rain ALS Care Center |
|  | 北京蝴蝶结结节性硬化症罕见病关爱中心 | TSC China Alliance |
|  | 北京康心马凡综合征关爱中心 | Beijing Kangxin Marfan Syndrome Care Center |
|  | 北京企鹅之家小脑萎缩症病患关爱中心 | China Spinocerebellar Ataxia Association |
|  | 北京市美儿脊髓性肌萎缩症关爱中心 | Meier Advocacy & Support Center for SMA |
|  | 北京血友之家罕见病关爱中心 | China Home of Hemophilia |
|  | 北京正宇粘多糖罕见病关爱中心 | Beijing Zhengyu MPS Disease Center |
|  | 北京至爱杜氏肌营养不良关爱中心 | Beijing Dear Dad and Mom Duchenne Muscular Dystrophy Care Centre |
|  | CAH互助之家 | Congenital adrenal hyperplasia (CAH) Peer Support Group |
|  | 瓷娃娃罕见病关爱中心 | China-Dolls Center for Rare Disorders |
|  | 多发性硬化之家 | Multiple Sclerosis Home |
|  | 高苯丙胺酸血症爱心传递联盟 | Hyperphenylalaninemia Love Transmission Alliance |
|  | FH关爱中心 | FH (Familial Hypercholesterolemia) Care Center |
|  | 戈谢病协会 | China Gaucher Disease Association |
|  | 肯尼迪罕见病关爱中心 | China Kennedy’s Disease Care Center |
|  | 朗格罕天使之家 | LCH Home of Angels |
|  | 蓝梅淋巴管肌瘤罕见病关爱中心 | LAM China |
|  | 老K之家 | Kallmann |
|  | NMO上海之家 | NMO Family Shanghai |
|  | 庞贝氏罕见病关爱中心 | China Pompe Care Center |
|  | 上海德博蝴蝶宝贝关爱中心 | Debra China |
|  | 上海浦东风信子亨廷顿舞蹈症关爱中心 | Huntington’s Disease China |
|  | 铜娃娃罕见病关爱中心 | Chinese Organization for Wilson's Disease |
|  | 硬皮病关爱之家 | Chinese Organization for Scleroderma |
|  | 月亮孩子之家 | Chinese Organization for Albinism |
|  | 浙江小胖威利罕见病关爱中心 | Prader-Willi Syndrome Care & Support Center |
|  | 中国法布雷病友会 | Fabry China |
|  | 中国尼曼匹克关爱中心 | China Niemann-Pick Disease Association |
|  | 卓蔚宝贝支持中心 | China Dravet Syndrome Patient Support Group |
