# Supplementary material for: Patterns of the Health and Economic Burden of 33 Rare Diseases in China: Nationwide Web-Based Study
Source: JMIR Public Health Surveill. 2024 Aug 27;10:e57353. doi: 10.2196/57353 (PMC11387910; doi:10.2196/57353)
Supplement: Multimedia Appendix 3 [file publichealth_v10i1e57353_app3.docx]

**Multimedia Appendix 3.** Short-Form Health Survey items and responses indicating some or severe problems.

| **Scales** | **Item** | **Subscale** | **Content** | **Response categories** | **Reponses indicating some/severe problems** |
| --- | --- | --- | --- | --- | --- |
| Physical Component Summary (PCS) | 1 | General Health | General health | Excellent/very good/good/fair/poor | Fair/poor |
|  | 2 | Physical Function | Moderate activities | Limited a lot/limited a little/not limited at all | Limited a lot/limited a little |
|  | 3 | Physical Function | Climb several flights of stairs | Limited a lot/limited a little/not limited at all | Limited a lot/limited a little |
|  | 4 | Role Physical | Accomplished less (physical) | All of the time/most of the time/some of the time/a little of the time/none of the time | All of the time/most of the time/some of the time |
|  | 5 | Role Physical | Limited in kind of work | All of the time/most of the time/some of the time/a little of the time/none of the time | All of the time/most of the time/some of the time |
|  | 8 | Role Emotional | Pain-interference | All of the time/most of the time/some of the time/a little of the time/none of the time | All of the time/most of the time/some of the time |
| Mental Component Summary (MCS) | 6 | Role Emotional | Accomplished less (emotional) | All of the time/most of the time/some of the time/a little of the time/none of the time | All of the time/most of the time/some of the time |
|  | 7 | Bodily Pain | Did work less carefully | Not at all/a little bit/moderately/quite a bit/extremely | All of the time/most of the time/some of the time |
|  | 9 | Mental Health | Calm and peaceful | All of the time/most of the time/some of the time/a little of the time/none of the time | Some of the time/a little of the time/none of the time |
|  | 10 | Vitality | Energy or vitality | All of the time/most of the time/some of the time/a little of the time/none of the time | Some of the time/a little of the time/none of the time |
|  | 11 | Mental Health | Downhearted and blue | All of the time/most of the time/some of the time/a little of the time/none of the time | All of the time/most of the time/some of the time |
|  | 12 | Social Function | Social limitations | All of the time/most of the time/some of the time/a little of the time/none of the time | All of the time/most of the time/some of the time |
