# Supplementary material for: Patterns of the Health and Economic Burden of 33 Rare Diseases in China: Nationwide Web-Based Study
Source: JMIR Public Health Surveill. 2024 Aug 27;10:e57353. doi: 10.2196/57353 (PMC11387910; doi:10.2196/57353)
Supplement: Multimedia Appendix 4 [file publichealth_v10i1e57353_app4.docx]

**Multimedia Appendix 4.** Demographic and disease-related characteristics among all participants (n=16,945).

| **Variable** | **n (%)** |
| --- | --- |
| **Age** (years), mean (SD) | 21.95 (17.85) |
| **Sex** |  |
| Male | 9486 (56.0%) |
| Female | 7459 (44.0%) |
| **Educational attainment** ^a^ |  |
| Below high school | 7027 (41.5%) |
| High school or above | 9877 (58.3%) |
| **Household registration** |  |
| Rural | 8505 (50.2%) |
| Urban or overseas | 8440 (49.8%) |
| **Poverty** |  |
| Non-poverty | 14410 (85.0%) |
| Poverty | 2535 (15.0%) |
| **Active treatment** |  |
| Non-active | 5283 (31.2%) |
| Active | 11662 (68.8%) |
| **Delay in diagnosis** |  |
| <1 year | 12929 (76.3%) |
| 1-2 years | 2170 (12.8%) |
| ≥3 years | 1610 (9.5%) |
| **Comorbidity** |  |
| None | 8398 (49.6%) |
| At least one | 8545 (50.4%) |
| **Disease duration**, mean (SD) | 6.83 (7.46) |
| **Recent diagnosis** |  |
| No | 14055 (82.9%) |
| Yes | 2890 (17.1%) |

^a^ parental education (at least one parent below high school, both parents with high school or above) for pediatric patients.
