# Supplementary material for: Patterns of the Health and Economic Burden of 33 Rare Diseases in China: Nationwide Web-Based Study
Source: JMIR Public Health Surveill. 2024 Aug 27;10:e57353. doi: 10.2196/57353 (PMC11387910; doi:10.2196/57353)
Supplement: Multimedia Appendix 5 [file publichealth_v10i1e57353_app5.docx]

**Multimedia Appendix 5.** Number of participants according to disease and ICD-10 codes.

| **Disease** | **Adult (n=8,454)** | **Pediatric (n=8,491)** | **Total (n=16,945)** |
| --- | --- | --- | --- |
| ***Diseases of the nervous system*** | | | |
| Myasthenia gravis (MG) | 2,066 | 237 | 2,303 |
| Duchenne Muscular Dystrophy (DMD) | 54 | 1279 | 1333 |
| Multiple sclerosis (MS) | 690 | 16 | 706 |
| Neuromyelitis Optica (NMO) | 303 | 15 | 318 |
| Dravet syndrome (DS) | 3 | 301 | 304 |
| Huntington's Disease (HD) | 247 | 3 | 250 |
| Kennedy Disease, Spinal and Bulbar Muscular Atrophy | 211 | 0 | 211 |
| Amyotrophic lateral sclerosis (ALS) | 188 | 0 | 188 |
| Spinocerebellar Ataxias | 157 | 0 | 157 |
| Spinal muscular atrophy (SMA) | 114 | 0 | 114 |
| ***Endocrine, nutritional and metabolic diseases*** | | | |
| Phenylketonuria | 56 | 2,280 | 2336 |
| Congenital adrenal hyperplasia (CAH) | 25 | 631 | 656 |
| Hepatolenticular degeneration (HLD); Wilson disease | 305 | 108 | 413 |
| Albinism | 105 | 226 | 331 |
| Mucopolysaccharidosis (MPS) | 22 | 226 | 248 |
| Fabry disease | 157 | 28 | 185 |
| Tetrahydrobiopterin deficiency | 3 | 169 | 172 |
| Kallmann syndrome, KS | 137 | 3 | 140 |
| Gaucher disease (GD) | 52 | 80 | 132 |
| Glycogen storage disease type (pompe disease) | 90 | 39 | 129 |
| Idiopathic hypogonadotropic hypogonadism (IHH) | 86 | 0 | 86 |
| Niemann-Pick disease (NPC) | 4 | 62 | 66 |
| Homozygous familial hypercholesterolemia (HoFH) | 16 | 8 | 24 |
| ***Congenital malformations, deformations and chromosomal abnormalities*** | | | |
| Tuberous sclerosis complex (TSC) | 159 | 808 | 967 |
| Marfan syndrome (MS) | 779 | 116 | 895 |
| Inherited epidermolysis bullosa (EB) | 182 | 324 | 506 |
| Prader-Willi Syndrome (PWS) | 10 | 436 | 446 |
| Osteogenesis imperfecta (OI) | 84 | 68 | 152 |
| ***Diseases of the blood and blood-forming organs and certain disorders involving the immune mechanism*** | | | |
| Hemophilia | 1009 | 761 | 1770 |
| ***Diseases of the musculoskeletal system and connective tissue*** | | | |
| Systemic sclerosis (SSc) | 821 | 176 | 997 |
| ***Neoplasms*** | | | |
| Lymphangioleiomyomatosis (LAM) | 250 | 0 | 250 |
| Langerhans cell histiocytosis (LCH) | 44 | 85 | 129 |
| ***Diseases of the circulatory system*** | | | |
| Idiopathic pulmonary arterial hypertension (IPAH) | 25 | 6 | 31 |
