# Supplementary material for: Patterns of the Health and Economic Burden of 33 Rare Diseases in China: Nationwide Web-Based Study
Source: JMIR Public Health Surveill. 2024 Aug 27;10:e57353. doi: 10.2196/57353 (PMC11387910; doi:10.2196/57353)
Supplement: Multimedia Appendix 6 [file publichealth_v10i1e57353_app6.docx]

**Multimedia Appendix 6.** Probability of reporting some or severe problems of physical and mental health items and the mean proportion of out-of-pocket expense categories relative to annual household income by clusters.

| **Variables** | **Overall** | **Cluster** | | |
| --- | --- | --- | --- | --- |
|  |  | **Overall low** | **Overall high** | **Extremely high** |
| **Adult patients** | | | | |
| *Health impact indicator ^a^* | *N (%)* | *Probability* | *Probability* | *Probability* |
| PH_1 | 6055 (71.6%) | 0.32 | 0.88 | 0.86 |
| PH_2 | 5512 (65.2%) | 0.20 | 0.83 | 0.91 |
| PH_3 | 6080 (71.9%) | 0.31 | 0.89 | 0.88 |
| PH_4 | 5921 (70.0%) | 0.18 | 0.91 | 0.83 |
| PH_5 | 6216 (73.5%) | 0.24 | 0.94 | 0.85 |
| PH_6 | 6042 (71.5%) | 0.28 | 0.89 | 0.90 |
| MH_1 | 5537 (65.5%) | 0.14 | 0.86 | 0.86 |
| MH_2 | 5488 (64.9%) | 0.17 | 0.84 | 0.87 |
| MH_3 | 4311 (51.0%) | 0.16 | 0.65 | 0.75 |
| MH_4 | 6319 (74.8%) | 0.37 | 0.90 | 0.90 |
| MH_5 | 5760 (68.1%) | 0.34 | 0.82 | 0.84 |
| MH_6 | 5823 (68.9%) | 0.19 | 0.89 | 0.86 |
| *Economic impact indicator ^b^* | *Mean (SD)* | *Mean proportion* | *Mean proportion* | *Mean proportion* |
| Direct medical cost | 105.12% (3.39) | 38.47% | 95.88% | 2460.81% |
| Direct non-medical cost | 40.82% (1.95) | 13.88% | 38.30% | 914.57% |
| Indirect cost | 20.98% (1.23) | 6.69% | 19.20% | 512.81% |
| **Pediatric patients** | | | | |
| *Health impact indicator ^a^* | *N (%)* | *Probability* | *Probability* | *Probability* |
| PH_1 | 3383 (39.8%) | 0.09 | 0.63 | 0.62 |
| PH_2 | 4655 (54.8%) | 0.18 | 0.74 | 0.82 |
| PH_3 | 5246 (61.8%) | 0.25 | 0.9 | 0.89 |
| PH_4 | 5043 (59.4%) | 0.23 | 0.68 | 0.86 |
| PH_5 | 4061 (47.8%) | 0.14 | 0.74 | 0.73 |
| PH_6 | 4308 (50.7%) | 0.15 | 0.79 | 0.77 |
| PH_7 | 4673 (55.0%) | 0.24 | 0.53 | 0.78 |
| PH_8 | 5716 (67.3%) | 0.36 | 0.68 | 0.91 |
| MH_1 | 5670 (66.8%) | 0.38 | 0.74 | 0.88 |
| MH_2 | 5279 (62.1%) | 0.31 | 0.58 | 0.86 |
| MH_3 | 6061 (71.4%) | 0.45 | 0.63 | 0.91 |
| MH_4 | 3943 (46.4%) | 0.19 | 0.58 | 0.67 |
| MH_5 | 4259 (50.1%) | 0.2 | 0.58 | 0.73 |
| MH_6 | 4795 (56.5%) | 0.38 | 0.74 | 0.88 |
| MH_7 | 4561 (53.7%) | 0.31 | 0.58 | 0.86 |
| MH_8 | 4307 (50.7%) | 0.45 | 0.63 | 0.91 |
| MH_9 | 5155 (60.7%) | 0.19 | 0.58 | 0.67 |
| MH_10 | 5233 (61.6%) | 0.2 | 0.58 | 0.73 |
| *Economic impact indicator ^b^* | *Mean (SD)* | *Mean proportion* | *Mean proportion* | *Mean proportion* |
| Direct medical cost | 103.22% (3.92) | 67.0% | 6475.9% | 105.2% |
| Direct non-medical cost | 45.30% (2.49) | 30.3% | 2518.5% | 46.7% |
| Indirect cost | 22.86% (1.38) | 16.3% | 592.8% | 25.5% |

^a^ items of quality of life instrument (SF-12 for adult patients, PedsQL for pediatric patients) reporting some/severe problem: PH: physical health; MH: mental health; ^b^ expense by different categories: yearly expenses divided by annual household income.
