# Supplementary material for: Patterns of the Health and Economic Burden of 33 Rare Diseases in China: Nationwide Web-Based Study
Source: JMIR Public Health Surveill. 2024 Aug 27;10:e57353. doi: 10.2196/57353 (PMC11387910; doi:10.2196/57353)
Supplement: Multimedia Appendix 7 [file publichealth_v10i1e57353_app7.docx]

**Multimedia Appendix 7.** Quality of life instrument scores and annual disease-related expenses (in US$) by clusters.

| **Variables** | **Overall** | **Cluster** | | |
| --- | --- | --- | --- | --- |
|  |  | **Overall low** | **Overall high** | **Extremely high** |
| **Adult patients** | *N (%)* | *Mean (SD)* | *Mean (SD)* | *Mean (SD)* |
| *SF-12 Physical Health* |  |  |  |  |
| Item 1 | 3.84 (0.97) | 2.95 (0.93) | 4.19 (0.72) | 4.16 (0.91) |
| Item 2 | 1.87 (0.74) | 1.22 (0.46) | 2.13 (0.67) | 2.27 (0.61) |
| Item 3 | 1.99 (0.75) | 1.34 (0.54) | 2.26 (0.65) | 2.27 (0.66) |
| Item 4 | 3.28 (1.33) | 1.89 (0.86) | 3.84 (1.03) | 3.79 (1.27) |
| Item 5 | 3.40 (1.33) | 2.0 (0.97) | 3.97 (1.00) | 3.88 (1.24) |
| Item 6 | 3.15 (1.20) | 2.05 (0.93) | 3.6 (1.00) | 3.80 (1.02) |
| Physical Component Summary ^a^ | 39.85 (10.14) | 50.12 (6.91) | 35.71 (8.10) | 35.83 (8.00) |
| *SF-12 Mental Health* |  |  |  |  |
| Item 1 | 3.08 (1.24) | 1.86 (0.73) | 3.56 (1.04) | 3.77 (1.13) |
| Item 2 | 3.06 (1.24) | 1.90 (0.76) | 3.52 (1.08) | 3.8 (1.10) |
| Item 3 | 2.67 (0.98) | 2.03 (0.83) | 2.92 (0.92) | 3.18 (1.04) |
| Item 4 | 3.31 (1.11) | 2.42 (0.92) | 3.66 (0.97) | 3.65 (0.99) |
| Item 5 | 3.08 (1.06) | 2.36 (0.83) | 3.36 (1.00) | 3.47 (1.12) |
| Item 6 | 3.22 (1.28) | 1.92 (0.85) | 3.74 (1.03) | 3.74 (1.12) |
| Mental Component Summary ^b^ | 40.13 (10.59) | 49.47 (7.49) | 36.40 (9.25) | 34.66 (9.42) |
| *Disease-related expenses (USD$)* | *Mean (SD)* | *Mean (SD)* | *Mean (SD)* | *Mean (SD)* |
| Direct medical cost ^c^ | 5040.7 (10502.3) | 3593.1 (7385.8) | 5106.2 (8918.3) | 39032.2 (48795.6) |
| Direct non-medical cost ^d^ | 1845.6 (6026.2) | 1280.3 (3554.1) | 1929.6 (5967.7) | 11347.1 (24790.0) |
| Indirect cost ^e^ | 931.1 (3407.5) | 577.9 (1971.4) | 1002.6 (3481.1) | 5642.0 (12603.3) |
| **Pediatric patients** | *N (%)* | *Mean (SD)* | *Mean (SD)* | *Mean (SD)* |
| *PedsQL Physical Functioning* |  |  |  |  |
| Item 1 | 2.3 (1.39) | 1.46 (0.93) | 2.92 (1.36) | 2.95 (1.43) |
| Item 2 | 2.8 (1.52) | 1.69 (1.05) | 3.63 (1.27) | 3.32 (1.38) |
| Item 3 | 2.99 (1.48) | 1.86 (1.09) | 3.83 (1.14) | 3.68 (1.29) |
| Item 4 | 2.98 (1.51) | 1.86 (1.07) | 3.82 (1.22) | 3.16 (1.68) |
| Item 5 | 2.60 (1.48) | 1.61 (1.00) | 3.32 (1.34) | 3.21 (1.51) |
| Item 6 | 2.60 (1.40) | 1.64 (0.93) | 3.32 (1.24) | 3.32 (1.20) |
| Item 7 | 2.48 (1.13) | 1.76 (0.87) | 3.02 (0.99) | 2.58 (1.17) |
| Item 8 | 2.80 (1.15) | 2.01 (0.94) | 3.39 (0.90) | 2.95 (1.31) |
| Physical Functioning Score ^f^ | 57.61 (28.23) | 81.61 (16.91) | 39.85 (20.83) | 46.38 (25.44) |
| *PedsQL Emotional Functioning* |  |  |  |  |
| Item 1 | 2.73 (1.07) | 2.06 (0.94) | 3.22 (0.87) | 3.00 (1.29) |
| Item 2 | 2.6 (1.03) | 1.93 (0.88) | 3.10 (0.82) | 2.74 (1.28) |
| Item 3 | 2.84 (1.05) | 2.19 (0.98) | 3.32 (0.82) | 2.68 (1.11) |
| Item 4 | 2.35 (1.10) | 1.71 (0.85) | 2.82 (1.03) | 2.47 (0.96) |
| Item 5 | 2.44 (1.15) | 1.74 (0.86) | 2.96 (1.06) | 2.63 (1.30) |
| Emotional Functioning Score ^g^ | 60.20 (22.53) | 76.8 (17.47) | 47.91 (17.37) | 57.37 (27.05) |
| *PedsQL Social Functioning* |  |  |  |  |
| Item 1 | 2.68 (1.26) | 1.79 (0.89) | 3.34 (1.07) | 2.89 (1.15) |
| Item 2 | 2.62 (1.26) | 1.71 (0.84) | 3.30 (1.09) | 2.79 (1.32) |
| Item 3 | 2.51 (1.20) | 1.67 (0.79) | 3.13 (1.06) | 2.63 (1.07) |
| Item 4 | 2.94 (1.45) | 1.75 (0.97) | 3.82 (1.07) | 2.95 (1.35) |
| Item 5 | 3.02 (1.47) | 1.81 (0.97) | 3.93 (1.07) | 2.84 (1.50) |
| Social Functioning Score ^h^ | 56.22 (29.36) | 81.42 (17.34) | 37.53 (21.42) | 54.21 (29.17) |
| *Disease-related expenses (USD$)* | *Mean (SD)* | *Mean proportion* | *Mean proportion* | *Mean proportion* |
| Direct medical cost | 5623.9 (13075.0) | 4366 (7438.5) | 6267.4 (13477.6) | 79764.4 (119333.5) |
| Direct non-medical cost | 2178.4 (6759.1) | 1630.1 (5927.1) | 2520.2 (6849.5) | 18770.9 (38088.0) |
| Indirect cost | 1195.4 (5748.5) | 911.8 (6389.4) | 1381 (5090.3) | 7545.4 (18358.2) |

^a^ Physical Component Summary score (39.85) lower than population norm of Hong Kong (50.2) [1] and Sichuan, China (51.2) [2].

^b^ Mental Component Summary score (40.13) lower than population norm of Hong Kong (50.1) [1] and Sichuan, China (49.9) [2].

^c^ Direct medical cost (US$ 5040.7) higher than Chinese patients with ischemic stroke (US$ 4258.0) [3] and type II diabetes (US$ 790.3) [4].

^d^ Direct non-medical cost (US$ 1845.6) higher than Chinese patients with ischemic stroke (US$ 446.0) [3].

^e^ Indirect cost (US$ 931.1) lower than Chinese patients with ischemic stroke (US$ 4506.8) [3].

^f^ Physical Functioning Score (57.61) lower than healthy Chinese children (86.33) [5].

^g^ Emotional Functioning Score (60.20) lower than healthy Chinese children (80.25) [5].

^h^ Social Functioning Score (56.22) lower than healthy Chinese children (88.16) [5].

References:

[1] Lam CL, Wong CK, Lam ET, et al. Population norm of Chinese (HK) SF-12 health survey_version 2 of Chinese adults in Hong Kong. 32.

[2] Li N, Liu D, Liu C, et al. Assessing quality of life in an urban population in Chengdu using the SF-12 [in Chinese]. *Sichuan Da Xue Xue Bao Yi Xue Ban = Journal of Sichuan University Medical Science Edition* 2010; 41: 1044–1046.

[3] Lv W, Wang A, Wang Q, et al. One-year direct and indirect costs of ischaemic stroke in China. *Stroke and Vascular Neurology* 2023; svn.

[4] Li X, Xu Z, Ji L, et al. Direct medical costs for patients with type 2 diabetes in 16 tertiary hospitals in urban China: A multicenter prospective cohort study. *Journal of Diabetes Investigation* 2019; 10: 539–551.

[5] Ji Y, Chen S, Li K, et al. Measuring health-related quality of life in children with cancer living in mainland China: feasibility, reliability and validity of the Chinese mandarin version of PedsQL 4.0 Generic Core Scales and 3.0 Cancer Module. *Health Qual Life Outcomes* 2011; 9: 103.
