# Supplementary material for: Patterns of the Health and Economic Burden of 33 Rare Diseases in China: Nationwide Web-Based Study
Source: JMIR Public Health Surveill. 2024 Aug 27;10:e57353. doi: 10.2196/57353 (PMC11387910; doi:10.2196/57353)
Supplement: Multimedia Appendix 8 [file publichealth_v10i1e57353_app8.docx]

**Multimedia Appendix 8.** Number of clusters determined with the elbow method: (A) adult patients; (B) pediatric patients.
