# Supplementary material for: Patterns of the Health and Economic Burden of 33 Rare Diseases in China: Nationwide Web-Based Study
Source: JMIR Public Health Surveill. 2024 Aug 27;10:e57353. doi: 10.2196/57353 (PMC11387910; doi:10.2196/57353)
Supplement: Multimedia Appendix 9 [file publichealth_v10i1e57353_app9.docx]

**Multimedia Appendix 9.** Silhouette coefficients and number of clusters by adult and pediatric patients.
